# Molecular Detection and Characterization of Tick-borne Pathogens in Dogs and Ticks from Nigeria

**DOI:** 10.1371/journal.pntd.0002108

**Published:** 2013-03-07

**Authors:** Joshua Kamani, Gad Baneth, Kosta Y. Mumcuoglu, Ndadilnasiya E. Waziri, Osnat Eyal, Yifat Guthmann, Shimon Harrus

**Affiliations:** 1 Parasitology Division, National Veterinary Research Institute, Vom, Nigeria; 2 Koret School of Veterinary Medicine, The Hebrew University of Jerusalem, Rehovot, Israel; 3 Department of Microbiology and Molecular Genetics, The Kuvin Center for the Study of Infectious and Tropical Diseases, Hebrew University–Hadassah Medical School, Jerusalem, Israel; 4 Central Diagnostic Division, National Veterinary Research Institute, Vom, Nigeria; University of California, Davis, United States of America

## Abstract

**Background:**

Only limited information is currently available on the prevalence of vector borne and zoonotic pathogens in dogs and ticks in Nigeria. The aim of this study was to use molecular techniques to detect and characterize vector borne pathogens in dogs and ticks from Nigeria.

**Methodology/Principal Findings:**

Blood samples and ticks (*Rhipicephalus sanguineus*, *Rhipicephalus turanicus* and *Heamaphysalis leachi*) collected from 181 dogs from Nigeria were molecularly screened for human and animal vector-borne pathogens by PCR and sequencing. DNA of *Hepatozoon canis* (41.4%), *Ehrlichia canis* (12.7%), *Rickettsia* spp. (8.8%), *Babesia rossi* (6.6%), *Anaplasma platys* (6.6%), *Babesia vogeli* (0.6%) and *Theileria* sp. (0.6%) was detected in the blood samples. DNA of *E. canis* (23.7%), *H. canis* (21.1%), *Rickettsia* spp. (10.5%), *Candidatus* Neoehrlichia mikurensis (5.3%) and *A. platys* (1.9%) was detected in 258 ticks collected from 42 of the 181 dogs. Co- infections with two pathogens were present in 37% of the dogs examined and one dog was co-infected with 3 pathogens. DNA of *Rickettsia conorii israelensis* was detected in one dog and *Rhipicephalus sanguineus* tick. DNA of another human pathogen, *Candidatus* N. mikurensis was detected in *Rhipicephalus sanguineus* and *Heamaphysalis leachi* ticks, and is the first description of *Candidatus* N. mikurensis in Africa. The *Theileria* sp. DNA detected in a local dog in this study had 98% sequence identity to *Theileria ovis* from sheep.

**Conclusions/Significance:**

The results of this study indicate that human and animal pathogens are abundant in dogs and their ticks in Nigeria and portray the potential high risk of human exposure to infection with these agents.

## Author Summary

In Nigeria, dogs are not only kept as pets, but are also used for hunting as well as a source of animal protein among some ethnic groups. Ticks are known to infest dogs and serve as vectors for some pathogens of zoonotic and veterinary importance. There is limited information on the prevalence and distribution of vector borne pathogens in dogs and ticks in Nigeria. The aim of the study was to detect and characterize vector borne pathogens in dogs and ticks from Nigeria using molecular methods. The results of this study showed a high estimate of vector borne pathogens in Nigerian dogs (77.3%) and ticks (63.3%). DNA of *Candidatus* N. mikurensis, an emerging pathogen of humans was detected in *Rhipicephalus sanguineus* and *Heamaphysalis leachi* ticks. Another human pathogen, *Rickettsia conorii israelensis* the causative agent of Mediterranean spotted fever was detected in *Rhipicephalus sanguineus* ticks. This is the first description of *Candidatus* N. mikurensis in Africa and *Rickettsia conorii israelensis* in Nigeria. These results indicate that the use of molecular techniques for the diagnosis of emerging infections in developing countries is of utmost importance in assisting physicians and veterinarians in making accurate diagnoses and providing the appropriate treatment for their patients.

## Introduction

Several tick-borne bacteria and parasites are important pathogens of humans and animals [1]. Being haematophagous, ticks are capable of transmitting disease agents such as viruses, bacteria and protozoa. Historically, they have been considered second only to mosquitoes in their ability to transmit disease agents [2]. Ticks attach to their hosts, facilitating transmission of infectious agents to the host and their spread to different geographical regions via traveling pets or other means of transportation [3]. Globalization and increased international trade, urbanization, climate change and increased travel and mobility of pets have resulted in rapid extension of the zoogeographical range for many tick species [1]. In areas where canine vector-borne diseases are endemic, dogs can be simultaneously or sequentially infected with more than one vector-borne agent [3], [4]. Because blood sucking vectors contain infected host blood and pathogens, they are reliable indicators for the existence of pathogens in a specific area [5]. Therefore, it is recommended to periodically screen animals and vectors for pathogen carriage.

Several molecular surveys have evaluated the existence of multiple vector borne pathogens in specific regions including, Europe [4], Middle East [6], Asia [7] and Africa [8]. Epidemiological surveillance of disease occurrence and prevalence is required to map local risk, to acquaint physicians and veterinarians with the prevalence of pathogens and emergence of new infectious agents and forecast possible vector-borne infection outbreaks. This can be achieved by the use of molecular diagnostic techniques, data analysis and mathematical models as well as veterinary clinical surveillance.

In Nigeria, the diagnosis of vector-borne pathogens (VBPs) is usually based on the microscopic detection of pathogens in peripheral blood smears, sometimes serology is employed and rarely molecular methods are used. Microscopic diagnosis may lack sensitivity and is time consuming while serology usually indicates exposure rather than active infection, and might mislead due to serological cross reactions with other closely related organisms. Conversely, molecular detection is more sensitive and specific. As data on canine vector-borne infections in Nigeria is scarce [9], [10], this study aimed at broadening the knowledge on these canine pathogens and their ectoparasites. The objective of this study was to molecularly detect and, characterize various vector-borne pathogens in dogs and ticks in four states of Nigeria.

## Materials and Methods

### Study area

**Figure 1:**
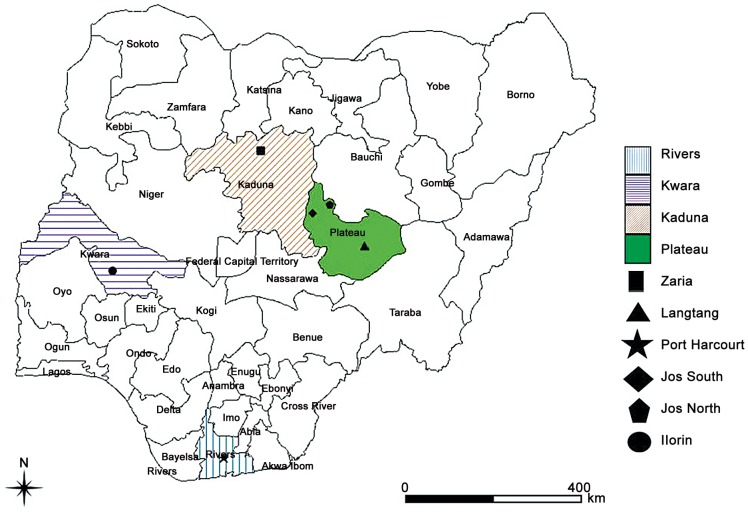
Map of Nigeria, West Africa showing states (shaded) where samples were collected. Legends describe the location of veterinary clinics and hospitals where samples were collected.

The study was conducted in the 4 Nigerian states; Plateau (9°10′N9°45′E), Kaduna (10°20′N7°45′E), Kwara (8°30′N 5°00′E) and Rivers (4°45′N 6°50′E) (Figure 1).

### Ethics statement

The study protocol was read and approved by The National Veterinary Research Institute Vom, Nigeria Ethical Committee on Animal Use and Care. Animals were treated in a humane manner and in accordance with authorizations and guidelines for Ethical Conduct in the Care and Use of Nonhuman Animals in Research of the American Psychological Association (APA) for use by scientists working with nonhuman animals (American Psychological Association Committee on Animal Research and Ethics in 2010).

### Sampling of dogs

One hundred and eighty one dogs from 4 states of Nigeria presented to private or government veterinary hospitals between August and December 2011 were selected. The selection criteria included dogs infested with ticks or manifesting clinical signs of tick borne diseases, such as anemia, weakness, pyrexia, anorexia and haemoglobinuria. Demographic data, signalment (age, sex, and breed) and clinical signs were recorded for each dog. Five ml of blood were collected from the cephalic or jugular vein into sterile EDTA tubes, and kept at 4°C until arrival at the laboratory.

### Sampling of ticks

Ticks were collected from dogs infested at the time of presentation into test tubes containing absolute ethanol and transported to the laboratory. Thereafter, samples were preserved at −20°C and transported in a cool box to Israel for identification and DNA analysis. A total of 258 ticks were collected from 42 domestic dogs. After identification, ticks from each dog were grouped according to their species. One to three ticks from each dog were pooled for analysis. Seventy six tick pools were processed for DNA extraction. Most of the ticks selected were partially or fully engorged adult females, nymphs and larvae.

### DNA extraction

#### Blood samples

DNA was extracted from EDTA-anticogulated blood using the Illustra blood genomic Prep Mini Spin kit (GE Healthcare UK Limited) according to the manufacturer's instructions. DNA concentrations were determined by measuring the absorbance at 260 nm (*A*_260_) with a NanoVue Spectrophotometer (GE Healthcare, UK Limited).

#### Ticks

Ticks were minced using a sterile scalpel blade before homogenization in sterile Eppendorf tubes containing 50 µl phosphate buffered saline (PBS). Total DNA was extracted from each tick pool using the Illustra tissue and cell genomic Prep Mini Spin kit (GE Healthcare UK Limited), adjusted in 100 µl of TE buffer and stored at −20°C until further use.

#### Real Time PCR for detection of *Ehrlichia* and *Anaplasma* spp

A 97 base pair segment of the 16S ribosomal RNA (16S rRNA) gene of *Ehrlichia canis* and *Anaplasma platys* was targeted using primers 16S-F and 16S-R as previously described [6]. DNA from *E. canis* 611 tissue culture was obtained from The Koret School of Veterinary Medicine, The Hebrew University of Jerusalem, Rehovot Israel and was used as a positive control. DNA from blood of a specific pathogen free (SPF) dog was used as a negative control. The negative and non-template controls (NTC) as well as the positive control were included in each reaction in duplicates. Non template control reactions were done using the same procedures and reagents described above but without DNA added to the PCR reaction to rule out PCR contaminations.

#### Real Time PCR for detection of *Rickettsia* spp

Initial detection of *Rickettsia* sp. was performed by screening all DNA samples for the presence of a 133-bp citrate synthase gene (*gltA*) fragment using primers rico173F and rico173R as previously described [11]. Positive samples from this reaction were further analyzed for the presence of a 178–189-bp fragment of the outer membrane protein A gene (*ompA*), using primers 107F and 299R as previously described [12]. DNA extracted from cultured *R. conorii israelensis* was used as a positive control, and two negative control samples containing all the ingredients of the reaction except DNA were used for all trials.

#### Conventional PCR assays for *Babesia* and *Hepatozoon* spp

A 408 base-pair fragment of the 18S ribosomal RNA (*18S rRNA*) gene of *Babesia* spp. was targeted using primers PIRO-A and PIRO-B as previously described [13]. For the detection of *Hepatozoon* spp., the forward primer HEP-F and the reverse primer HEP-R were used as previously reported targeting a 666 bp fragment of the *18S rRNA* gene [14], [15].

Positive controls of naturally infected dogs with *Babesia vogeli* (Bab 36799) and *Hepatozoon canis* (HEP 7423), as well as negative DNA controls from colony-bred dogs negative by PCR for vector-borne pathogens were run with each corresponding PCR reaction. Non-template control (NTC) reactions were done using the same procedures and reagents described above but without DNA added to the PCR reaction to rule out contaminations. PCR was performed using Syntezza PCR-Ready High Specificity (Syntezza Bioscience, Israel). Amplification was performed using a programmable conventional thermocycler (Biometra, Goettingen, Germany).

PCR products were electrophoresed on 1.5% agarose gels stained with ethidium bromide and checked under UV light for the size of amplified fragments by comparison to a 50 bp DNA molecular weight marker.

#### Sequencing

DNA amplicons from positive samples were purified (EXOSAP-IT, USB, Cleveland, Ohio, USA) and sequenced using forward primers at the Center for Genomics Technologies, Hebrew University of Jerusalem, Israel. DNA sequences obtained were evaluated with Chromas Lite software version 2.01(Technelysium pty Ltd) and compared for similarity to sequences deposited in GenBank, using the BLAST program hosted by NCBI, National Institutes of Health, Bethesda, MD USA http://www.ncbi.nlm.nih.gov/BLAST. Selected sequences were deposited in GenBank.

#### Statistics

Data generated in the study were analyzed by a χ^2^ test using the Statistix 8 software. Association of pathogen DNA between dogs and ticks was computed using the Medcal statistical software [16]. P values <0.05 were considered significant.

## Results

### Pathogen DNA in dog blood

A total of 181 dogs were examined during this study, 66 (36.5%) being males and 102 (56.4%) were females. No information on sex was available for 13 (7.2%) of the samples analyzed (Table 1). The age ranges of the study population were 0–6 months, 32%; 7–12 months, 30.9%; 13–24 months, 19.9%; 25–36 months, 3.9%; >36 months, 5%, while no information on age was available for 15 (8.3%) dogs. Seventy eight (43.1%) of the dogs studied belonged to a local (Nigerian) breed, 61 (33.7%) belonged to pure foreign breeds, 27 (14.9%) were of cross- breeds while no information on breed was available for 15 (8.3%) dogs. The majority of the sampled dogs were from Plateau State (150; 82.9%), followed by Rivers State (17; 9.4%), Kaduna State (11; 6.1%) and Kwara State (3; 1.7%).

**Table 1:** Distribution of vector-borne pathogens among dog population studied.

Variables	No. (%) examined	No. (%) positive for								Total
		*E. canis*	*A. platys*	*B. rossi*	*B. vogeli*	*Theileria* sp.	*H. canis*	*Rickettsia* spp.		
								*gltA*	*OmpA*	
**Sex**										
Male	66 (36.5)	4 (6.1)	5 (7.6)	3 (4.5)	0 (0)	0 (0)	27 (40.9)	5 (7.6)	1 (1.5)	44 (24.3)
Female	102 (56.4)	11 (10.8)	7 (6.9)	9 (8.8)	0 (0)	1 (0.9)	38 (37.3)	9 (8.8)	0 (0)	75 (41.4)
DNA	13 (7.2)	8 (61.5)	0 (0)	0 (0)	1 (1.7)	0 (0)	10 (76.9)	2 (15.3)	0 (0)	21 (11.6)
**Age group (months)**										
0–6	58 (32.0)	7 (12.1)	3 (5.2)	5 (8.6)	0 (0)	0 (0)	21 (36.2)	6 (10.3)	0 (0)	42 (22.7)
7–12	56 (30.9)	7 (12.5)	4 (7.1)	4 (7.1)	0 (0)	0 (0)	24 (42.9)	2 (3.6)	0 (0)	41 (22.1)
13–24	36 (19.9)	2 (5.6)	5 (13.9)	1 (2.8)	0 (0)	0 (0)	15 (41.7)	5 (13.9)	1 (2.8)	28 (15.5)
25–36	7 (3.9)	0 (0)	0 (0)	1 (14.3)	0 (0)	1 (14.3)	1 (14.3)	0 (0.0)	0 (0)	3 (1.7)
>36	9 (5.0)	0 (0)	0 (0)	0 (0)	0 (0)	0 (0)	2 (22.2)	1 (11.1)	0 (0)	3 (1.7)
DNA	15 (8.3)	7 (46.7)	0 (0)	1 (6.7)	1 (6.7)	0 (0)	12 (80.0)	2 (13.3)	0 (0)	23 (12.7)
**Breed**										
Local	78 (43.1)	7 (9.0)	5 (6.4)	5 (5.1)	0 (0)	0 (0)	35 (44.9)	9 (11.5)	1 (1.3)	61 (33.1)
Exotic	61 (33.7)	6 (9.8)	6 (9.8)	6 (9.8)	0 (0)	1 (1.6)	16 (26.2)	4 (6.6)	0 (0)	39 (215)
Cross	27 (14.9)	3 (11.1)	1 (3.7)	1 (7.4)	0 (0)	0 (0)	12 (44.4)	1 (3.7)	0 (0)	18 (9.9)
DNA	15 (8.3)	7 (46.7)	0 (0)	0 (0)	1 (6.7)	0 (0)	12 (80.0)	2 (13.3)	0 (0)	22 (12.2)
**Sampling Area**										
Rivers state	17 (9.4)	2 (11.8)	2 (11.8)	2 (11.8)	0 (0)	0 (0)	2 (11.8)	0 (0)	0 (0)	8 (4.4)
Kwara state	3 (1.7)	0 (0)	1 (33.3)	0 (0)	0 (0)	0 (0)	0 (0)	0 (0)	0 (0)	1 (0.6)
Kaduna state	11 (6.1)	7 (63.3)	0 (0)	0 (0)	1 (9.1)	0 (0)	9 (81.8)	2 (18.2)	0 (0)	19 (10.5)
Plateau State										
a. Jos North	41 (22.7)	7 (17.1)	4 (9.8)	6 (14.6)	0 (0)	0 (0)	11 (26.8)	2 (4.9)	0 (0)	30 (16.0)
b. Jos South	84 (46.4)	6 (7.1)	4 (4.8)	4 (4.8)	0 (0)	1 (1.2)	30 (35.7)	10 (11.9)	1 (1.2)	55 (30.4)
c. Lantang North	25 (13.8)	1 (4.0)	1 (4.0)	0 (0)	0 (0)	0 (0)	23 (92.0)	2 (8.0)	0 (0)	27 (14.9)
**Total**	**181 (100)**	**23 (12.7)**	**12 (6.6)**	**12 (6.6)**	**1 (0.6)**	**1 (0.6)**	**75 (41.4)**	**16 (8.8)**	**1 (0.6)**	**140 (77.3)**

DNA = Data not available; *E. canis = Ehrlichia canis*; *A. platys = Anaplasma platys*; *B. rossi = Babesia rossi*; *B. vogeli = Babesia vogeli*; *H. canis = Hepatozoon canis*.

Overall estimate of infection with VBPs was 77.3% (140/181) in sampled dogs. Single infections occurred in 73(40.3%) dogs while co- infection with more than one pathogen occurred in 67 (37%) of the dogs examined. Co- infections with *H. canis* were most prevalent (14.4%) followed by *E. canis* and *Rickettsia* spp. (6.6%) each (Table 2). Single infections occurred mostly in dogs within the age range of 9–12 months. Co-infections were mostly detected in dogs between 2–12 months of age.

**Table 2:** Number of dogs infected with single or multiple vector- borne pathogens.

Nature of infection	Pathogen species detected in dogs							
	*E. canis*	*A. platys*	*B. rossi*	*B. vogeli*	*Theileria* sp.	*H. canis*	*Rickettsia* sp.	Total (%)
Single infection	10	6	6	0	1	46	4	73 (40.3)
Co infection with								
*E. canis*	-	0	1	0	0	10	1	12 (6.6)
*A. platys*	0	-	1	0	0	5	2	8 (4.4)
*B. rossi*	1	1	-	0	0	4	1	7 (3.9)
*B. vogeli*	1	0	0	-	0	1	-	2 (1.1)
*Theileria* sp.	0	0	0	0	0	0	0	0(0)
*H. canis*	10	4	3	1	0	-	8	26 (14.4)
*Rickettsia* sp.	1	1	1	0	0	9	-	12 (6.6)
**Total**	**23**	**12**	**12**	**1**	**1**	**75**	**16**	**140 (77)**

*E. canis = Ehrlichia canis*; *A. platys = Anaplasma platys*; *B. rossi = Babesia rossi*; *B. vogeli = Babesia vogeli*; *H. canis = Hepatozoon canis*.

*Hepatozoon canis* was the most frequently detected pathogen in dogs (41.4%), followed by *E. canis*, *Rickettsia* spp., *Babesia rossi* and *A. platys* (12.7%, 8.8%, 6.6% and 6.6% respectively). *Babesia vogeli*, *Theileria* sp. and *R. conorii israelensis* were detected in one dog each (Table 1). There was no significant difference (p>0.05) in prevalence of these pathogens between the various groups of dogs studied (Table 1). Sequences of pathogens derived from dog's blood in this study were deposited in GenBank under the following accession numbers; *H. canis* (JQ976620–JQ976629); *B. rossi* (JQ976603–JQ976616); *B. vogeli* (JQ976617); *Theileria* sp. (JQ976619); *E. canis* (JQ976630–JQ976641) and *A. platys* (JQ976642–JQ976653).

### Pathogen DNA in ticks

A total of 258 ticks (128 adults, 124 nymphs and 6 larvae) partially or fully engorged belonging to two genera *Rhipicephalus* and *Haemaphysalis*, removed from 42 dogs were examined for various VBPs. *Ehrlichia canis*, *H. canis* and *Rickettsia* spp. DNA were detected in *R. sanguineus*, *R. turanicus* and *H. leachi* ticks. NA of various VBPs was detected in all the different tick species examined in this study. A total of 76 tick pools were tested out of which 18 (23.7%), 16(21.1%), 8(10.5%) and 4(5.3%) were positive for the DNA of *E. canis*, *H. canis*, *Rickettsia* spp. and *Candidatus* N. mikurensis respectively, while *A. platys* and *R. conorii israelensis* DNA were detected in one tick pool each (Table 3). Sequences from ticks were assigned the following accession numbers: *H. canis* (JX027010–JX027020), *Candidatus* N. mikurensis (JX027021–JX027024), *E. canis* (JQ976654–JQ976665), *A. platys* (JQ976666) and *R. conorii israelensis* (JX259321 and JX259322).

**Table 3:** Comparison of DNA sequence similarities between pathogens detected in dogs and ticks in this study and GenBank deposited sequences.

Pathogen sequences from dog blood		Pathogen sequences from ticks	
Pathogen genotype (No. positive)-Accession No.	First Genbank Match Accession No. (% sequence similarity)	Pathogen genotype (No. positive)- Accession No.	First GenBank Match Accession No. (% sequence similarity)
***Babesia*** ** spp.**			
*B. rossi* (1) –JQ976615	*Babesia rossi* -AB303074.1 (100)	-	-
*B. rossi* (9) - JQ976603	*Babesia rossi* -AB303074.1 (99)	-	-
*B. rossi* (2) –JQ976612	*Babesia rossi* -AB303074.1 (98)	-	-
*B. vogeli* (1) -JQ976617	Uncultured *Babesia* clone seqBCV79-JN717134.1 (99)	-	-
***Theileria*** ** sp.**			
*Theileria* sp (1)-JQ976622	*Theileria ovis* -GU726904.1 (98)	-	-
***Ehrlichia*** ** spp.**			
*E. canis* (17) - JQ976631	Uncultured *Ehrlichia* sp. clone -JQ260861 (100)	*E. canis* (15) - JQ976654	Uncultured *Ehrlichia* spp -JQ260861 (100)
*E. canis* (2)- JQ976630	Uncultured *Ehrlichia* sp. clone-JQ260861 (99)	*E. canis* (2) –JQ976659	*E. chaffensis*-JQ085940.1 (99)
*E. canis* (3) - JQ976639	Uncultured *Ehrlichia* sp. clone-JQ260861 (97)		
		***Candidatus*** ** Neoehrlichia mikurensis**	
		*C. N mikurensis* (4) - JX027021	*C.N. mikurensis*- JQ359051 (100)
***Anaplasma*** ** spp.**			
*A. platys* (8) - JQ976650	*Anaplasma platys* isolate A.pl -JQ 396431(100)	*A. platys* (1) - JQ976666	*Anaplasma platys* -JQ396431 (99)
*A. platys* (1) –JQ976643	*Anaplasma platys* isolate A.pl-JQ 396431 (99)		
*A.platys* (1) - JQ976648	*Anaplasma platys* isolate A.pl-JQ 396431 (97)		
*Anaplasma* sp (1)-JQ976642	uncultured Anaplasmataceae bacterium-JN581373.1 (99)		
***Hepatozoon canis***			
*H. canis* (6) –JQ976617	*Hepatozoon canis* -DQ 111754 (99)	*H. canis* (2)-JX027013	*Hepatozoon canis* -DQ111754 (100)
*H. canis* (3) JQ976626	*Hepatozoon canis* -EU289222 (99)	*H. canis* (5)-JX027011	*Hepatozoon canis* -DQ111754 (99)
*H. canis* (1)-JQ976629	*Hepatozoon canis* -JF 459994 (99)	*H. canis* (3) - JX027010	*Hepatozoon canis*-GU376457 (99)
***Rickettsia*** ** spp.**			
*Rickettsia* sp (*gltA*) (16) JX259323	Uncultured *Rickettsia* sp JQ664729 (100)	*Rickettsia* sp (*gltA*) (6) JX259324	Uncultured *Rickettsia* sp JQ664729 (100)
*R. c. israelensis* (1) JX259321	*R. c. israelensis* (EU122392 (100)	*R. c. israelensis* (1) JX259322	*R. c. israelensis* EU122392 (100)

*E. canis = Ehrlichia canis*; *A. platys = Anaplasma platys*; *B. rossi = Babesia rossi*; *B. vogeli = Babesia vogeli*; *H. canis = Hepatozoon canis*; *E. chaffeensis = Ehrlichia chaffeensis*; *C.* N mikurensis* = Candidatus* N. mikurensis; *R. c. israelensis = Rickettsia. conorii israelensis*.

### Comparison between the presence of pathogen DNA in blood and ticks from the same dog

Pathogen DNA as single or multiple infections were detected in 26/76 (34.2%) tick pools removed from dogs with tick infestation at the time of clinical presentation and sampling. Blood and ticks from 7 dogs (nos. 1, 8, 33, 34, 37, 38 and 39) were free from DNA of the various VBPs tested for in this study. DNA of pathogens was detected in ticks removed from 7 other dogs (nos. 3, 4, 12, 18, 25, 31 and 36) but none was detected in the blood of their dog host. Conversely, DNA of various pathogens was detected in 7 dogs (nos. 5,7,13, 16, 28, 29 and 49), but no pathogen DNA was detected in ticks removed from them. Ticks collected from 4 dog (nos. 2, 15, 22 and 23) as well as the dogs from which they were removed were both positive for *E. canis* DNA. Similarly, DNA of *H. canis* was detected in 3 dogs (nos. 20, 21 and 24) and ticks removed from each of them. However, DNA of *H. canis* only was detected in 3 dogs (nos. 11, 35 and 42) but DNA of both *H. canis* and *E. canis* was detected in ticks removed from them. Different pathogen's DNA was detected in 10 dogs (nos. 6, 9, 10 14, 17, 19, 26, 27, 35 and 40) as compared to the ticks removed from them. Nine (11.8%) of the tick pools were co-infected by two or more pathogens. There was a significant association between *H. canis* DNA in dogs and ticks removed from them (Relative risk = 2.69; 95% Confidence Interval = 1.2–5.8; Z = 2.51; p = 0.012). However, there was no significant association between the detection of pathogen DNA in dogs blood and ticks removed from them for *E. canis*, (RR = 1.56; 95% CI = 0.44–5.45; Z = 0.69; p = 0.49), *A. platys* (RR = 0.59; 95% CI = 0.025–14.3; Z = 0.32; p = 0.75) or *B. rossi* (RR = 0.07; 95% CI = 0.004–1.13; Z = 1.88; p = 0.061). *Babesia* spp. and *Theileria* spp. DNA were not detected in any of the tick pools tested.

### Identity of pathogen sequences amplified from dogs and ticks

*Babesia rossi* and *B. vogeli* sequences from this study had 98–100% and 99% similarities, respectively, with the first matched BLAST result from GenBank (Table 3), while *Theileria* sp. had 98% sequence similarity with *Theileria ovis*. *Ehrlichia canis* sequences from blood of dogs and ticks in this study had 97–100% and 100% similarities, respectively, with the first matched BLAST result from GenBank. Two sequences had 99% similarity with *Ehrlichia chaffeensis* as the first GenBank match from BLAST. However, attempts to validate the identity of this species by PCR for secretory genes (*SodB/VirB 3*, *VirB 4*, and *VirB 9*) did not yield confirmatory results. Four sequences from ticks had 100% sequence identity to *Candidatus* N. mikurensis. *Anaplasma platys* sequences from dogs and ticks had 97–100% and 99% sequences similarities, respectively, with the first matched BLAST result from GenBank. Similarly, *H. canis* sequences from dogs and ticks had 99% and 99–100% similarities, respectively, with *H. canis* sequences deposited in GenBank (Table 3).

The rickettsial *gltA* gene fragment was detected in 16 of 181 (8.8%) dog blood samples and in 8 of 76 (10.5%) tick pools examined. Rickettsial *ompA* DNA was found in one (0.6%) blood and one tick sample. Both sequences were identical and were 100% similar to *ompA* fragment from *R. conorii israelensis*. All the sequences detected in this study from dogs and the ticks removed from each of them were highly identical to each other (99–100%) for all the pathogens identified (Table 3).

## Discussion

Ticks and other haematophagous arthropods play a major role in the epidemiology of diseases of humans and animals globally. Their distribution and abundance determines the epidemiology of vector borne infections. The results of this study provide molecular evidence for the presence of *E. canis, H. canis, A. platys, B. rossi, B. vogeli, Theileria* sp., closely related to *T. ovis*, *Candidatus* N. mikurensis and *R. conorii israelensis* in dogs and ticks from Nigeria. DNA of at least one vector borne pathogen was detected in 77% of the dogs and 45% of the tick pools examined.

This is the first report documenting the identification of *Candidatus* N. mikurensis, *R. conorii israeliensis*, *A. platys* and *Theileria* sp. in dogs and ticks from Nigeria. In fact, it is the first detection of the zoonotic pathogen, *Candidatus* N. mikurensis, in Africa. *Candidatus* N. mikurensis is an emerging pathogen first described in 2004 affecting humans and animals with varying clinical manifestation [17]. This pathogen was reported in several hosts including *Ixodes* spp. ticks, *Rattus norvegicus*, humans and dogs from Japan, Switzerland and Germany [17]–[19]. Although *Ixodes* ticks of medical and veterinary importance are not found in Nigeria, *R. norvegicus* are common and serve as small mammal hosts to multi- host ticks during their life cycle. As engorged ticks were screened in this study, it is possible that *R. sanguineus* ticks acquired *Candidatus* N. mikurensis from infected *R. norvegicus* or dogs. Due to the fact that this agent is a potential threat to humans, physicians should consider this pathogen in their differential diagnosis list in complicated unexplained fever of unknown etiology cases in Nigeria.

*Rickettsia gltA* DNA was detected in 8.8% and 10.5% of dogs and ticks respectively in this study. These estimates are almost similar to the 7.8% previously reported [9] but lower than the 20.6% infection rate reported for *R. africae* in ticks collected from cattle and vegetation in Nigeria [10]. Another report of prevalence of 0.4% *R. conorii* and 94–100% *R. africae* in *Rhipicephalus evertsi* was made in Guinea and Liberia, West Africa [20]. In the present study, 8.8% of blood samples and 10.5% of tick pools were positive for the rickettsial *gltA* but only one blood sample (0.6%) and one *R. sanguineus* tick pool (1.3%) were found positive for the rickettsial *ompA* gene, and their sequences were 100% identical to *R. conorii israelensis*. This is the first report indicating the presence of the agent of Mediterranean Spotted Fever in Nigeria. Rickettsiae with *ompA* gene are considered to be pathogenic, while those who exclude this gene are probably non-pathogenic endosymbionts [21]. *Rickettsia africae*, the etiologic agent of African tick fever in humans has been detected in ticks from Nigeria [9], [10] and other West African countries [20] but not in our study. It is possible *R. sanguineus*, *R. turanicus* and *H. leachi* found on dogs in this study are not competent vectors for this organism [20].

The detection of *A. platys* infection in Nigeria is also reported for the first time in this study. The estimate of 6.6% infection rate in dogs in this study is higher than the 4% reported for dogs in Italy [22], but lower than 16% reported in Venezuela [23]. *Anaplasma platys* is a thrombocytotropic bacteria of dogs that causes canine infectious cyclic thrombocytopaenia characterized by clinical abnormalities such as fever, anorexia, petechial haemorrhages, and uveitis [24].

*Theileria* sp. with 98% sequence similarity to *T. ovis* from a sheep in Iran [25] was detected in one dog in this study (Table 3). *Theileria* spp. have been reported in dogs from South Africa [26] and Spain [27]. The *Theileria* sp. in this study appears to be molecularly different from the previously described species.

The high estimate of *H. canis* (41.4%) and *E. canis* (12.7%) infections reported in dog blood in this study are higher than the 22% and 5%, respectively reported earlier in Zaria-Nigeria using microscopic examination of blood smears [28]. However, the estimate of 6.6% for *B. rossi* in this study is lower than previous reports of 10.2% [29] and 11.0% [30], and higher than 2.0% for dogs in Nigeria [28], but close to the 9.0% reported in Sudan [8]. Similarly, the estimate of 41.4% *H. canis* infection in this study is higher than the 20.3% previously reported in dogs from Nigeria, but almost similar to the 42.3% reported in Sudan [8]. The higher estimate of *H. canis* DNA in this study can be attributed to the sensitivity of the techniques used, enabling the detection of *E. canis* and *H. canis* at low bacterial and parasite loads. *Babesia canis* and *B. gibsoni* were not detected in any of the samples tested. This finding is in agreement with earlier molecular surveys in dogs from Nigeria [30] and can be attributed to the absence of their tick vectors in Nigeria. Although a case of *B. rossi* and *B. canis* co-infection in a local Nigerian dog that never left the country has been reported [31], the source of that infection could not be elucidated.

One recent study in Nigeria did not detect *Ehrlichia* spp. in *R. sanguineus* ticks [9], while another study reported a prevalence of 5.7% *Ehrlichia* spp. in ticks collected from cattle [10] which is much lower than the 23.7% detected in this study. The difference in prevalence may be attributed to variation in techniques used and source of samples.

Dogs are competent reservoir- hosts of several zoonotic pathogens and are infested by many blood-feeding arthropods. The role of ticks as vectors of these pathogens can be asserted considering the high sequence similarities (99–100%) between the pathogens detected from the host and those detected in ticks removed from them (Table 3). Of the 18 tick pools positive for *E. canis*, and 16 positive for *H. canis*, 22% and 38% of the pools were from *E. canis* and *H. canis* positive hosts, respectively. There was a significant association between the detection of *H. canis* DNA in dogs and ticks removed from the same dog, but no association was found for *E. canis*, *A. platys* or *B. rossi*. As all ticks were removed from dogs while they were attached and most of them were partially or fully engorged, it is impossible to ascertain whether the ticks were fed with infected blood or if they served as vectors and transmitted the pathogen to their present host. Considering the fact that the tick species included in this study have multi- host life cycle, they could have acquired infection during feeding on a previous infected host and transmitted the infection during their next feeding.

Dog breeding is a lucrative business in Nigeria, where dogs are used for trade and security. Dogs also serve as a food source and their meat is considered as a delicacy among some ethnic groups in Nigeria. These can expose humans directly or indirectly to zoonotic agents during handling of dogs and ticks carrying pathogens, or during processing and consumption of their meat. Further investigation is required to elucidate the role of ticks and the effect of these pathogens in causing diseases in humans in Nigeria.

In conclusion, this study has confirmed that several vector borne pathogens of humans and animals are highly prevalent in Nigeria and West Africa where the incidence of tick-borne infections appears to be underestimated. Physicians and veterinarians should be aware of the existence of these pathogens in Nigeria and should include them in the differential diagnoses for clinical illnesses with compatible clinical signs. Screening targeted groups for VBPs as well as humans with fever of unknown origin or undiagnosed cases for infection with *R. conorii israelensis* and *Candidatus* N. mikurensis is recommended.

## References

[1] Shaw SE, Michael JD, Richard JB, Breitschwerdt EB (2001) Tick-borne infectious diseases of dogs. Trends Parasitol 17: 74–80. 11228013 10.1016/s1471-4922(00)01856-0

[2] Hillyard PD (1996) Diseases carried by ticks in NW Europe: their medical and veterinary importance. In: Barnes, RSK, Coles, JH, eds. Ticks of North-West Europe. ynopses of the British Fauna (New Series). FSC Publications, 22–23

[3] Otranto D, Dantas-Torres F, Breitschwerdt EB (2009) Managing canine vector borne diseases of zoonotic concern: part one. Trends Parasitol 25: 157–163. 19269898 10.1016/j.pt.2009.01.003

[4] Cardoso L, Yisaschar-Mekuzas Y, Rodrigues FT, Costa Á, Machado J, et al (2010) Canine babesiosis in northern Portugal and molecular characterization of vector-borne co-infections. Parasite and Vectors 3: 27. 10.1186/1756-3305-3-27 PMC2865458 20377861

[5] Roux V, Raoult D (1999) Body lice as tools for diagnosis and surveillance of reemerging diseases. J. Clin. Microbiol 37: 596–599. 10.1128/jcm.37.3.596-599.1999 PMC84482 9986818

[6] Peleg O, Baneth G, Eyal O, Inbar J, Harrus S (2010) Multiplex real-time qPCR for the detection of *Ehrlichia canis* and *Babesia canis vogeli* . Vet Parasitol 173: 292–299. 20674177 10.1016/j.vetpar.2010.06.039

[7] Suksawat J, Xuejie Y, Hancock SI, Hegarty BC, Nilumhang P (2001) Serologic and molecular evidence of co infection with multiple vector borne pathogens in dogs from Thailand. J Vet Int Med 15: 453–462. 10.1892/0891-6640(2001)015<0453:sameoc>2.3.co;2 11596732

[8] Oyamada M, Bernard D, Mickae B, Jacques D, Bruno B, et al (2005) Detection of *Babesia canis rossi*, *B. canis vogeli*, and *Hepatozoon canis* in dogs in a village of Eastern Sudan by using a screening PCR and sequencing methodologies. Clin Diagn Lab Immunol 1343–1346. 16275954 10.1128/CDLI.12.11.1343-1346.2005 PMC1287771

[9] Ogo NI, de Mera IG, Galindo RC, Okubanjo OO, Inuwa HM, et al (2012) Molecular identification of tick-borne pathogens in Nigerian ticks. Vet Parasitol 187: 572–577. 22326937 10.1016/j.vetpar.2012.01.029

[10] Reye AL, Arinola OG, Hübschen JM, Muller CP (2012) Pathogen prevalence in ticks collected from the vegetation and livestock in Nigeria. Appl Environ Microbiol 78: 2562–2568. 22327584 10.1128/AEM.06686-11 PMC3318832

[11] Harrus S, Perlman-Avrahami A, Mumcuoglu KY, Morick D, Baneth G (2011) Molecular detection of *Rickettsia massiliae*, *Rickettsia sibirica mongolitimonae* and *Rickettsia conorii israelensis* in ticks from Israel. Clin Microbiol Infect 17: 176–180. 20331680 10.1111/j.1469-0691.2010.03224.x

[12] Kidd L, Maggi R, Diniz PPVP, Hegarty B, Tucker M, et al (2008) Evaluation of conventional and real-time PCR assays for detection and differentiation of Spotted Fever Group Rickettsia in dog blood. Vet Microbiol 129: 294–303. 18226476 10.1016/j.vetmic.2007.11.035

[13] Olmeda AS, Armstrong PM, Rosenthal BM, Valladares B, del Castillo A, et al (1997) A subtropical case of human babesiosis. Acta Trop 229–234. 10.1016/s0001-706x(97)00045-4 9241387

[14] Inokuma H, Okuda M, Ohno K, Shimoda K, Onishi T (2002) Analysis of the 18SrRNA gene sequence of a *Hepatozoon* detected in two Japanese dogs. Vet Parasitol 106: 265–271. 12062514 10.1016/s0304-4017(02)00065-1

[15] Rubini AS, Paduan KS, Cavalcante GG, Ribolla PE, O'Dwyer LH (2005) Molecular identification and characterization of canine *Hepatozoon* species from Brazil. Parasitol Res 97: 91–93. 15948009 10.1007/s00436-005-1383-x

[16] Medcalc Version 9.6.4.0 (2008) Available: http://www.medcalc.be. accessed on 13^th^ May, 2010.

[17] Kawahara M, Rikihisa Y, Isogai E, Takahashi M, Misumi H, et al (2004) Ultrastructure and phylogenetic analysis of ‘*Candidatus* Neoehrlichia mikurensis’ in the family Anaplasmataceae, isolated from wild rats and found in *Ixodes ovatus* ticks. Int J Syst Evol Microbiol 54 (Pt 5) 1837–1843. 15388752 10.1099/ijs.0.63260-0

[18] Elena L, Luce B, Christèle D, Lise G (2012) Infections and coinfections of questing *Ixodes ricinus* ticks by emerging zoonotic pathogens in Western Switzerland. Appl Environ Microbiol 78: 4606. 22522688 10.1128/AEM.07961-11 PMC3370488

[19] Diniz PPVP, Schulz BS, Hartmann K, Breitschwerdt EB (2011) “*Candidatus* Neoehrlichia mikurensis” infection in a dog from Germany. J Clin Microbiol 2059–2062. 21367991 10.1128/JCM.02327-10 PMC3122698

[20] Mediannikov O, Diatta G, Fenollar F, Sokhna C, Trape J-F, et al (2010) Tick-Borne Rickettsioses, Neglected Emerging Diseases in Rural Senegal. PLoS Negl Trop Dis 4: e821 doi:10.1371/journal.pntd.0000821. 20856858 10.1371/journal.pntd.0000821 PMC2939048

[21] Foongladda S, Inthawong D, Kositanont U, Gaywee J (2011) *Rickettsia*, *Ehrlichia*, *Anaplasma*, and *Bartonella* in Ticks and Fleas from Dogs and Cats in Bangkok. Vector-Borne Zoon Dis 11: 1336–1341. 10.1089/vbz.2010.0174 21612535

[22] de la Fuente J, Alessandra T, Victoria N Silviane N, Angelina A, et al (2006) Molecular characterization of *Anaplasma platys* strains from dogs in Sicily, Italy. Vet Res 2: 24. 10.1186/1746-6148-2-24 PMC1550391 16872489

[23] Huang H, Ahmet U, Miriam JP, Nelson GO, Yasuko R (2005) Prevalence and Molecular Analysis of *Anaplasma platys* in Dogs in Lara, Venezuela. Braz J Microbiol 36: 211–216.

[24] Ulutas B, Bayramli G, Karagenc T (2007) First case of *Anaplasma* (*Ehrlichia*) *platys* infection in a dog in Turkey. Turk J Vet Anim Sci 31 (4) 279–282.

[25] Zaeemi M, Haddadzadeh H, Khazraiinia P, Kazemi B, Bandehpour M (2011) Identification of different *Theileria* species (*Theileria lestoquardi*, *Theileria ovis*, and *Theileria annulata*) in naturally infected sheep using nested PCR-RFLP. Parasitol Res 108: 837–843. 20978792 10.1007/s00436-010-2119-0

[26] Matjila PT, Leisewitz AL, Oosthuizen MC, Jongejan F, Penzhorn BL (2008c) Detection of a *Theileria* species in dogs in South Africa. Vet Parasitol 157: 34–40. 18687528 10.1016/j.vetpar.2008.06.025

[27] Dixit P, Alok K, Dixit J, Varshney P (2010) Evidence of new pathogenic *Theileria* species in dogs. J Parasit Dis 34: 29–32. 21526030 10.1007/s12639-010-0009-0 PMC3081695

[28] Ezekoli CD, Ogunkoya AB, Abdullahi R, Tekedek LB, Sannusi A, et al (1983) Clinical and epidemiological studies on canine hepatozoonosis in Zaria, Nigeria. J Small Anim Pract 24: 455–460.

[29] Amuta EU, Atu BO, Houmsou RS, Ayashar JG (2010) *Rhipicephalus sanguineus* infestation and *Babesia canis* infection among domestic dogs in Makurdi, Benue State-Nigeria. Intl J Acadc Res 2/3: 170–172.

[30] Sasaki M, Olutayo O, Morito T, Kaisaku O, Aya M, et al (2007) Molecular survey of *Babesia canis* in dogs in Nigeria. J Vet Med Sci 69: 1191–1193. 18057838 10.1292/jvms.69.1191

[31] Kamani J, Sannusi A, Dogo AG, Tanko TJ, Egwu OK, et al (2010) *Babesia canis* and *Babesia rossi* co-infection in an untraveled Nigerian dog. Vet Parasitol 173: 334–335. 20705395 10.1016/j.vetpar.2010.06.040

